# Granulomatosis With Polyangiitis Complicated by Diffuse Alveolar Hemorrhage and Mitral Valve Chordal Rupture: An Uncommon Cardiopulmonary Manifestation

**DOI:** 10.7759/cureus.87618

**Published:** 2025-07-09

**Authors:** Ahmed R Fadel, Vipul Mayank, Sudhir Lohani

**Affiliations:** 1 Respiratory Medicine, Dartford and Gravesham NHS Trust, Dartford, GBR; 2 General Medicine, Dartford and Gravesham NHS Trust, Dartford, GBR

**Keywords:** anca-associated vasculitis, avacopan, cardiac involvement in vasculitis, diffuse alveolar hemorrhage, granulomatosis with polyangiitis (gpa), mitral valve chordal rupture, multisystem vasculitis, pr3-anca, rituximab (rtx), steroid-sparing therapy

## Abstract

Granulomatosis with polyangiitis (GPA) is an anti-neutrophil cytoplasmic antibodies (ANCA)-associated vasculitis that typically affects the upper and lower respiratory tract and kidneys. Cardiac complications, particularly those involving the valvular or subvalvular structures, are rare. We present the case of a 59-year-old woman with known anti-proteinase 3 (PR3)-ANCA-positive GPA who developed a severe disease flare characterized by diffuse alveolar hemorrhage (DAH) and mitral valve insufficiency due to chordae tendineae rupture. The diagnosis was confirmed by high-resolution computed tomography demonstrating ground-glass opacities consistent with DAH, markedly elevated PR3-ANCA titers, and echocardiography showing severe mitral regurgitation with ruptured chordae. She was treated with high-dose corticosteroids, rituximab, and avacopan, which resulted in marked clinical and radiological improvements. This case highlights the need for increased awareness of rare GPA manifestations, importance of multidisciplinary evaluation, and potential benefits of early immunosuppressive therapy.

## Introduction

Granulomatosis with polyangiitis (GPA) is a necrotizing vasculitis of small- to medium-sized vessels associated with anti-neutrophil cytoplasmic antibodies (ANCAs), most commonly anti-proteinase 3 (PR3). GPA predominantly affects the upper and lower respiratory tracts and kidneys; however, cardiac involvement is much less frequently recognized. At present, cardiac diseases can lead to substantial morbidity. Treatment is guided by disease severity and typically involves corticosteroids, rituximab, cyclophosphamide, and avacopan, a steroid-sparing C5a receptor antagonist [1,2]. Plasmapheresis may be considered in cases of refractory pulmonary or renal involvement [3].

This case report describes a rare presentation of GPA characterized by a combination of diffuse alveolar hemorrhage (DAH) and mitral valve chordae tendineae rupture. This association is exceptionally uncommon, underscoring the importance of considering cardiac ischemic complications in GPA, and the need for prompt recognition, diagnostic confirmation, and multidisciplinary management to improve outcomes.

## Case presentation

A 59-year-old woman with biopsy-proven GPA diagnosed 10 years ago had a history of chronic sinusitis and positive PR3-ANCA serology. She presented with one month of progressive shortness of breath and dry cough, followed by two weeks of low-grade fever and mild hemoptysis. Her previous course was complicated by left main bronchus occlusion, resulting in total collapse of the left lung. She remained in remission for over a decade prior to this presentation, without long-term immunosuppressive medications.

On admission, she was hypoxic (SpO₂ 88% on air). Examination revealed bibasal crackles and a pansystolic murmur. Laboratory tests showed a hemoglobin level of 90 g/L, white blood cell count of 22 × 10⁹/L, C-reactive protein (CRP) of 89 mg/L, erythrocyte sedimentation rate (ESR) of 70 mm/h, and procalcitonin of 0.12 ng/mL. Empirical treatment for community-acquired pneumonia was initiated with ceftriaxone.

**Figure 1:**
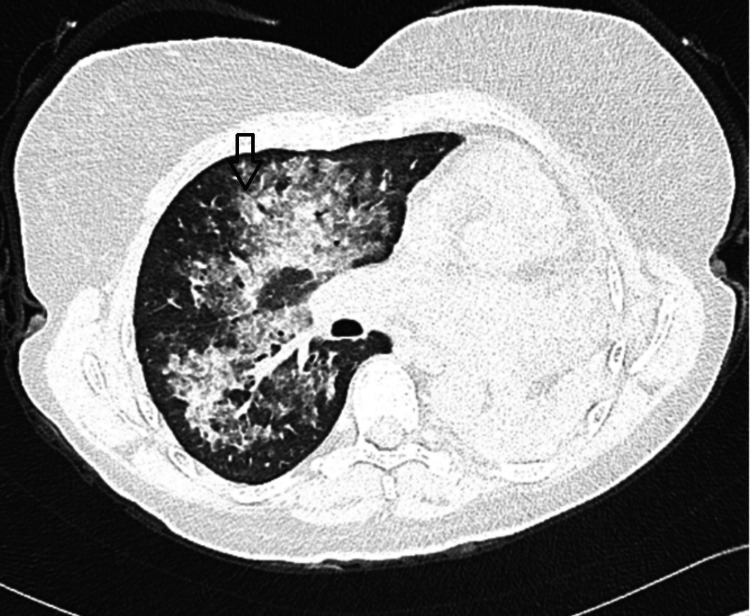
High-resolution computed tomography demonstrating right-sided ground-glass opacities (arrow) with subpleural sparing suggestive of DAH, a severe GPA complication with long standing total left lung collapse due to GPA-induced left main stem bronchus narrowing. Ground-glass opacities (i.e., “hazy areas reflecting alveolar filling”). DAH – diffuse alveolar hemorrhage, GPA – granulomatosis with polyangiitis

High-resolution chest computed tomography revealed right-sided ground-glass changes with subpleural sparing (Figure 1). Respiratory team input raised suspicion of DAH, especially in the context of marked eosinophilia (4.0 × 10⁹/L) and a significantly elevated PR3-ANCA titer (162 U/mL). Further workup showed autoimmune screening results consistent with vasculitic activity (Table 1).

**Table 1:** Extended autoimmune and infectious causes workup. ESR – erythrocyte sedimentation rate, ANA – antinuclear antibody, dsDNA – double-stranded DNA, glomerular BM – glomerular basement membrane, C3 – complement component 3, C4 – complement component 4, CCP – cyclic citrullinated peptide, PR3 – proteinase 3, serum galactomannan – serum test for fungal infection biomarker, B-D-Glucan – fungal cell wall component marker, EBV – Epstein-Barr virus, CMV – cytomegalovirus, Ig – immunoglobulin

Investigation	Result	Reference Value (Normal Range)	Interpretation
ESR	70 mm/hr	0-10mm/hr	Elevated
Rheumatoid factor	492 IU/ML	<14 IU/mL	Highly elevated
Antinuclear antibody	1:80 (Speckled pattern)		Weak positive
dsDNA antibody	Negative	Negative	Normal
Glomerular basement membrane antibody	Negative	Negative	Normal
C3 complement	1.72 g/L	0.90–1.80 g/L	Normal
C4 complement	0.30 g/L	0.10–0.40 g/L	Normal
CCP antibody	Negative	Negative	Normal
Anti-PR3 antibodies	124 IU/mL rising to 162 IU/mL	<20 IU/mL	Elevated, active disease
Serum Galactomannan	Negative	Negative	Normal
B-D-Glucan	Negative	Negative	Normal
EBV IgG, IgM	IgG positive, IgM negative	Negative, Negative	Previous infection
CMV	IgG Negative, IgM negative	Negative, Negative	Normal
Extended respiratory viral screen	Negative	Negative	Normal

Urine dipstick examination revealed hematuria and proteinuria. The urine protein-to-creatinine ratio was 48 mg/mmol. Renal function was preserved during the initial admission but later showed a transient decline following contrast exposure. Renal ultrasound revealed normal-sized kidneys with preserved corticomedullary differentiation.

**Figure 2:**
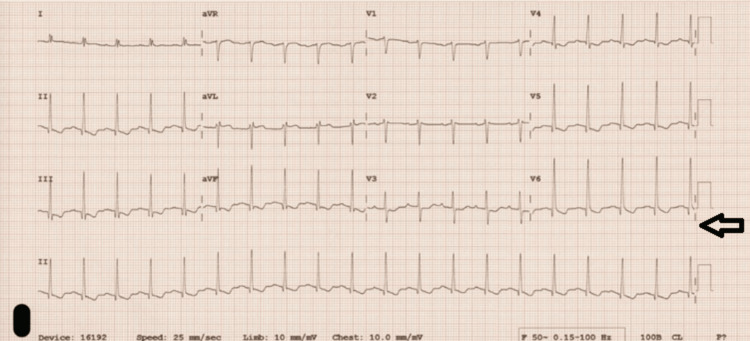
ECG: Inferolateral T-wave inversion in leads V4-V6-aVF and leads I-III.

**Figure 3:**
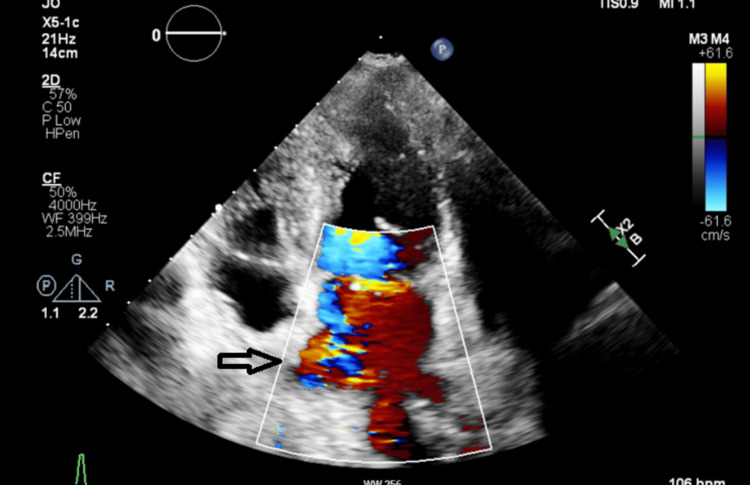
Transthoracic echocardiography: severe mitral valve regurgitation.

ECG showed lateral T-wave inversions (Figure 2), and troponin levels peaked at 186 ng/L, before decreasing to 18 ng/L in the absence of chest pain. Transthoracic echocardiography revealed severe mitral regurgitation and two mobile echogenic structures on the mitral valve leaflets (Figure 3). Infective endocarditis was considered; however, blood cultures were negative and there was no peripheral stigmata of infective endocarditis. Transoesophageal echocardiography was performed to exclude infective endocarditis and confirm that the echogenic structures dangling from the mitral valve leaflets represented ruptured chordae tendineae, with no evidence of vegetation. This finding confirmed the presence of severe mitral valve regurgitation.

The patient was deemed unfit for bronchoscopy because of severe mitral valve regurgitation and hypoxemia. Therefore, a diagnosis of diffuse alveolar hemorrhage was established based on the combination of clinical presentation, high-resolution CT imaging, and markedly elevated anti-PR3 ANCA titers, consistent with a GPA flare. In addition, the lack of fever spikes, multiple negative blood cultures, and negative procalcitonin results effectively excluded infection.

Intravenous methylprednisolone was initiated, and she was transferred to a tertiary centre for further management.

At the tertiary center, pulmonary function testing demonstrated a restrictive defect, with elevated carbon monoxide transfer coefficient (KCO) suggestive of diffuse alveolar hemorrhage (Table 2). Coronary angiography revealed non-obstructive coronary disease.

**Table 2:** Pulmonary function test results demonstrating restrictive pattern and elevated gas transfer coefficient consistent with diffuse alveolar hemorrhage. FEV1 – forced expiratory volume in the first second, FVC – forced vital capacity, VC – vital capacity, TLC – total lung capacity, RV – residual volume, TLCO – transfer factor for carbon monoxide, KCO – carbon monoxide transfer coefficient

Parameter	Measured Value	Reference Range (LLN–ULN)	% Predicted	Interpretation
Spirometry				
FEV1 (L)	1.21	1.80–2.92	51%	Decreased – Severe airflow limitation, consistent with total left lung collapse
FVC (L)	1.31	2.27–3.74	44%	Decreased – Severe restriction/volume loss
FEV1/FVC (%)	93	68–90		Increased – Consistent with restrictive pattern
VC (L)	1.40	2.54–3.91	43%	Decreased – Reduced lung capacity
Lung Volumes				
TLC (L)	2.40	3.89–5.83	51%	Decreased – Restrictive deficit
RV (L)	1.00	0.92–2.42	65%	Mildly decreased
RV/TLC (%)	42	18–42		Upper limit of normal
Gas Transfer (Corrected)				
TLCO corr (SI)	4.76	4.87–8.13	77%	Mildly decreased – Mild diffusion impairment
KCO (Hb) (SI)	2.16		147%	Elevated – Suggestive of alveolar hemorrhage

**Figure 4:**
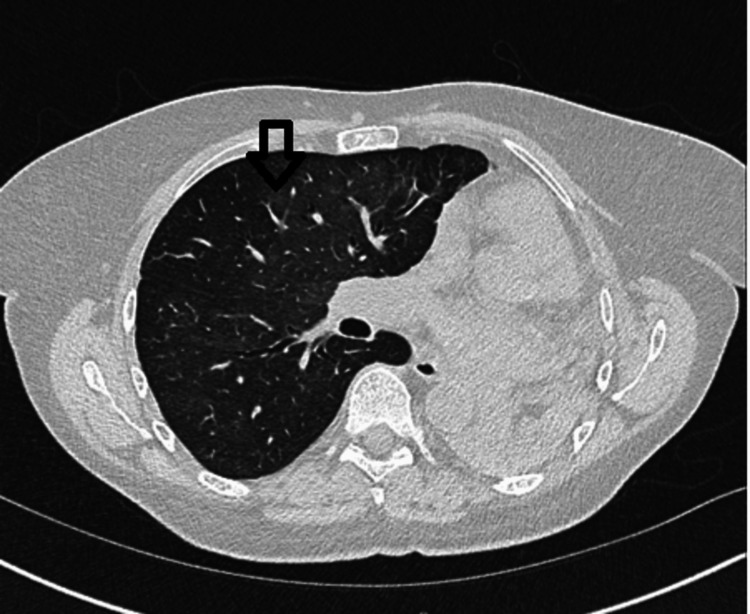
High-resolution computed tomography performed two weeks after initiation of high-dose steroids and rituximab, showing marked improvement in ground-glass changes in the right lung (arrow).

The patient was administered intravenous methylprednisolone, rituximab, and oral avacopan. Co-trimoxazole prophylaxis and a steroid tapering regimen were initiated. Inflammatory markers normalized, respiratory status improved, and she showed clinical and biochemical recovery. The key laboratory and imaging results supporting the diagnosis of diffuse alveolar hemorrhage and mitral valve chordae tendineae rupture are presented in Table 3. A follow-up high-resolution chest CT scan demonstrated a significant improvement in the previously noted peribronchovascular ground-glass opacification in the right lung (Figure 4), confirming the radiological response to treatment.

**Table 3:** Summary of laboratory and imaging findings supporting the diagnosis of GPA flare, diffuse alveolar hemorrhage, and mitral valve chordal rupture. DAH – diffuse alveolar hemorrhage, GPA – granulomatosis with polyangiitis, CRP – C-reactive protein, ESR – erythrocyte sedimentation rate, PR3 – proteinase 3, ANCA – anti-neutrophil cytoplasmic antibodies, ANA – antinuclear antibody, HRCT – high-resolution computed tomography

Domain	Findings	Interpretation
Hematology	Hemoglobin: 90 g/L WBC: 22 × 10⁹/L Platelets: Normal	Anemia consistent with DAH; inflammatory respons
Inflammatory Markers	CRP: 89 mg/L ESR: 70 mm/hr Procalcitonin: 0.12 ng/mL	Elevated inflammatory markers; low procalcitonin made sepsis less likely
Autoimmune Serology	PR3-ANCA: Increased to 162 U/mL (from prior remission) ANA: Weak positive (1:80 speckled) Rheumatoid Factor: Highly elevated (492 IU/mL)	Active vasculitis flare (GPA relapse)
Renal Parameters	Urine dipstick: Hematuria & proteinuria Creatinine: Mild transient increase post-contrast	Renal vasculitis involvement
Initial HRCT Chest	Right-sided ground-glass opacities with subpleural sparing	Radiologic confirmation of DAH
Echocardiography	Severe mitral regurgitation Mobile echogenic structures (ruptured chordae)	Structural valve rupture confirmed
Follow-up HRCT Chest	Resolution of ground-glass opacities after immunosuppression	Radiographic response to therapy
Coronary Angiography	Non-obstructive coronary disease	Excludes atherosclerotic cause

She was discharged with rheumatology and interstitial lung disease (ILD) follow-up for ongoing biological therapy and steroid tapering. Although she had severe mitral regurgitation, she did not show any significant manifestations of heart failure or pulmonary edema; therefore, she was scheduled for elective mitral valve replacement.

## Discussion

GPA is a rare form of ANCA-associated vasculitis characterized by granulomatous inflammation and necrotizing vasculitis of the small- and medium-sized blood vessels [1]. Its pathophysiology involves both innate and adaptive immune responses, notably the production of antibodies against proteinase 3 (PR3-ANCA) or myeloperoxidase (MPO-ANCA). PR3-ANCA plays a major role in GPA by promoting neutrophil activation, endothelial injury, and the formation of neutrophil extracellular traps (NETs), which contribute to vascular inflammation and tissue damage [2].

The disease predominantly affects the upper and lower respiratory tracts and kidneys but may involve virtually any organ. Clinical manifestations include nasal crusting, sinusitis, pulmonary nodules, alveolar hemorrhage, and glomerulonephritis. Less commonly, GPA presents with cardiac, gastrointestinal, neurological, or cutaneous involvements. Cardiac manifestations, while underreported, are significant contributors to morbidity and may precede or accompany systemic symptoms [4].

In a systematic review, DAH was assessed as a rare but severe pulmonary manifestation of GPA. Patients were predominantly male (59%), with an average age of approximately 49 years. Nearly all patients presented with anemia, and 61.5% had hemoptysis, as observed in our patient, although its absence did not exclude DAH. Two-thirds of patients had renal impairment, reflecting high disease activity. Mechanical ventilation is frequently needed, with approximately 30-65% of patients requiring invasive support across cohorts [5].

The PEXIVAS trial and guidelines of the European Alliance of Associations for Rheumatology (EULAR) highlight the central role of corticosteroids and rituximab in managing these patients, reserving plasmapheresis for life-threatening or refractory cases. Bronchoalveolar lavage demonstrating hemosiderin-laden macrophages remains the diagnostic standard, although CT findings and the clinical context often suffice. In our case, radiographic evidence and eosinophilia supported the diagnosis, despite the absence of bronchoalveolar lavage [6,7].

Treatment primarily included high-dose steroids, cyclophosphamide, and rituximab. Over time, rituximab use increased, driven by the RAVE trial showing its non-inferiority to cyclophosphamide, especially in relapsing disease. Plasmapheresis was used in 71% of the patients despite more recent evidence from Walsh et al.’s randomized control trial and the 2021 American College of Rheumatology (ACR) guidelines demonstrating no survival benefit. Accordingly, plasma exchange is now mainly recommended as a salvage therapy for refractory diseases. Extracorporeal membrane oxygenation (ECMO) is occasionally employed in critically ill patients who do not respond to conventional ventilation.

Imaging typically demonstrated bilateral ground-glass opacities, and bronchoscopy confirmed DAH in many cases. Differential diagnoses included pulmonary edema, infection, and other autoimmune vasculitides.

Mortality varied across studies, ranging from 15% to 36%. Case reports uniformly reported survival, likely reflecting publication bias [5-7].

Myocardial ischemia in GPA is an under-recognized but clinically significant manifestation that can arise from vasculitis affecting the small- and medium-sized coronary arteries. Unlike classic atherosclerosis, ischemia in GPA is primarily driven by necrotizing vasculitis, granulomatous inflammation, and immune-mediated endothelial injury [6]. Inflammation can lead to intimal damage, mural thrombus formation, or complete vessel occlusion. Even when the coronary angiography findings appear normal, autopsy studies have confirmed that coronary arteritis in patients with GPA may be widespread and clinically silent, sometimes involving bypass grafts, resulting in myocardial infarction without preceding symptoms. As shown in a case reported by Dewan et al. [7], imaging revealed extensive soft tissue thickening around the native coronary arteries and grafts, and histopathology confirmed granulomatous capillaritis. Myeloperoxidase-positive neutrophils and leukocytoclasias were key histological features. Ischemia may present with ECG changes, elevated troponin levels, or syncope mimicking acute coronary syndromes. Noninvasive imaging modalities, such as contrast-enhanced cardiac MRI or CT angiography, can detect perivascular inflammation; however, biopsy remains the diagnostic gold standard. Prompt recognition and immunosuppressive therapy, including corticosteroids and rituximab, may lead to significant regression of coronary inflammation and symptom resolution [8,9].

Valvular involvement in GPA is rare, but clinically significant. Although GPA typically affects the respiratory tract and kidneys, cardiac manifestations, including valvular disease, are increasingly being recognized, particularly in autopsy studies. The most common valvular pathology is aortic regurgitation; however, mitral regurgitation, stenosis, and multivalvular involvement have also been reported. These lesions may result from granulomatous inflammation, leaflet thickening, perforation, or sterile vegetation, and often mimic infective endocarditis. In most cases, valvular damage progresses despite immunosuppression, necessitating surgical intervention [10].

Aortic valve involvement has been increasingly recognized in the literature. Histopathologically confirmed cases demonstrate characteristic features, such as granulomatous inflammation, geographic necrosis, and polymorphous microabscesses within valve tissue, which are pathognomonic of GPA rather than secondary to infection or degenerative disease. In some patients, valve dysfunction is caused by direct granulomatous infiltration and necrosis of the cusps or annulus, leading to leaflet thickening, erosion, and regurgitation. Importantly, vegetation is typically absent, and cultures are negative, distinguishing GPA-related valvular lesions from infective endocarditis [11]. Long-term outcomes depend on early recognition and surgical intervention, as indicated, along with systemic immunosuppression [12].

Mitral valve disease is particularly unusual, with only a few cases documented in the literature. Histopathological findings typically reveal granulomatous inflammation with necrotizing vasculitis affecting the valvular tissue, chordae tendineae, and the surrounding structures. Clinical manifestations may range from asymptomatic regurgitation to severe valvular dysfunction requiring surgical intervention. Early recognition of mitral involvement is essential, as delayed treatment may lead to irreversible structural damage or complications such as heart failure or embolic events [13-15].

Rupture of the chordae tendineae is an exceptionally rare but serious cardiac complication of GPA that typically arises from coronary vasculitis-induced ischemia. In the case described by Kawada et al. [16], a 75-year-old woman with GPA developed severe mitral regurgitation due to rupture of the posteromedial papillary muscle despite no evidence of obstructive coronary artery disease on angiography. Pathological analysis of the excised muscle revealed acute ischemic changes, including myocyte degeneration and interstitial hemorrhage. Similarly, Sahinoğlu et al. [17] reported a patient with GPA who developed rupture of the mitral chordae tendineae in association with multiple rapidly progressing solid and cavitary pulmonary nodules and splenic infarctions, reflecting widespread systemic vasculitis. Together, these cases highlight that valvular rupture can occur alongside systemic vascular involvement and may present as sudden mitral regurgitation, hemodynamic instability, or systemic embolic events.

These observations support a vasculitic mechanism affecting small- to medium-sized coronary vessels, which may lead to myocardial infarction and structural rupture, even when conventional angiography appears normal [8]. In addition to vasculitic injury, it is important to recognize that GPA is associated with an increased risk of acute coronary syndromes (ACS), as demonstrated in a Korean single-center cohort study reporting an incidence of ACS of 2.7% in patients with ANCA-associated vasculitis, which is significantly higher than that in the general Korean adult population (0.5-0.6%) [18].

Finally, infective endocarditis also needs to be carefully considered and excluded, as it is one of the leading causes of chordae tendineae rupture [19], as was done in our case as well as in the case described by Sahinoğlu et al. Echocardiography remains a crucial diagnostic tool for detecting subvalvular involvement, particularly in the absence of classic ischemic markers. This emphasizes the importance of considering cardiac ischemic complications, including papillary muscle or chordal rupture, in patients with GPA who develop acute valvular dysfunction and elevated cardiac biomarker levels, without angiographic findings.

Management

The management of GPA is divided into two key phases: induction and maintenance of remission. According to recent ACR and EULAR updates, GPA cases should be stratified as either organ/life-threatening or non-organ/life-threatening to guide treatment decisions [2].

In cases of organ- or life-threatening diseases, such as our patient’s presentation with diffuse alveolar hemorrhage, renal involvement, and cardiac complications, remission induction typically involves high-dose glucocorticoids in combination with either rituximab or cyclophosphamide. Rituximab is preferred in relapsing disease owing to its superior tolerability and non-inferiority in efficacy, as demonstrated in the RAVE trial [20].

In our case, rituximab was initiated along with oral avacopan, a novel oral C5a receptor inhibitor that blocks neutrophil migration and activation. Avacopan offers the benefit of reducing glucocorticoid-associated toxicity without compromising efficacy. The ADVOCATE trial demonstrated that avacopan was non-inferior to prednisone in inducing remission in ANCA-associated vasculitis and superior in sustaining remission at 52 weeks. Patients who received avacopan showed improved renal function, fewer serious adverse events, and reduced steroid exposure. These findings support its role as a valuable steroid-sparing agent in both induction and maintenance therapies, particularly for patients at a higher risk of glucocorticoid complications [21,22].

EULAR guidelines recommend starting oral prednisolone at 0.5-1 mg/kg/day, tapered to 5 mg daily over four to five months. Our patient received intravenous methylprednisolone followed by a tapering regimen in accordance with this protocol. Additionally, co-trimoxazole prophylaxis was prescribed, as recommended for patients receiving rituximab or high-dose steroids, owing to the increased risk of Pneumocystis jirovecii pneumonia (PJP) [7].

Plasma exchange (PLEX) is generally reserved for patients with a creatinine level ≥300 µmol/L or for those experiencing refractory pulmonary hemorrhage. However, the PEXIVAS trial found no significant mortality benefit and noted an increased risk of infection, thus limiting its routine use [3,6].

To maintain remission, rituximab remains the preferred agent, outperforming azathioprine in preventing relapse, as demonstrated in the MAINRITSAN trial [23]. Alternatives such as methotrexate or azathioprine can be considered in patients with adequate renal function (eGFR >60 mL/min/1.73m²). Maintenance therapy is typically continued for 24-48 months, with longer durations advised for relapsing disease or PR3-ANCA positivity [2,22].

Treatment decisions should be clinically driven rather than solely based on ANCA titer or B-cell counts. Immunoglobulin monitoring is essential during rituximab therapy and prophylactic strategies should be employed to prevent opportunistic infections.

In this case, the patient's management closely aligned with contemporary guidelines, involving rituximab, avacopan, glucocorticoid tapering, and infection prophylaxis. Continued multidisciplinary follow-up, including rheumatology and ILD input, is essential for monitoring disease activity and coordinating elective mitral valve repair.

Long-term management of GPA requires maintenance immunosuppression, surveillance for relapse, and multispecialty collaboration. Relapse is more common in PR3-positive patients and may present with pulmonary or renal involvement. Our patient’s follow-up includes rheumatology and ILD clinic support, with plans for elective mitral valve repair

Given the multisystem nature of GPA, differential diagnoses are broad and include other forms of ANCA-associated vasculitis (e.g., microscopic polyangiitis), systemic lupus erythematosus, infective endocarditis, and malignancies. Comprehensive autoimmune and infectious screening is essential for establishing a diagnosis, assessing disease activity, and excluding mimics. Our patient's negative cultures, imaging findings, and serological profile were consistent with active GPA.

## Conclusions

This case highlights the critical need to recognize diverse and potentially life-threatening manifestations of GPA. While GPA most frequently involves the upper respiratory tract, lungs, and kidneys, this report illustrates that cardiac complications such as mitral valve chordal rupture can emerge unexpectedly and coexist with severe pulmonary involvement, including DAH. These uncommon presentations underscore the importance of maintaining a high index of suspicion for vasculitic cardiac injury even in the absence of overt coronary artery disease on angiography. Multidisciplinary collaboration between respiratory physicians, cardiologists, rheumatologists, and intensive care specialists is essential to facilitate prompt diagnosis and early initiation of immunosuppressive therapy.

More broadly, this case underlines how evolving treatment strategies, such as incorporating avacopan as a steroid-sparing agent along with rituximab, can offer opportunities to improve disease control while reducing corticosteroid exposure. However, the optimal management approach for severe cardiac involvement in patients with GPA remains unclear. Future research should prioritize studies that clarify the prevalence, mechanisms, and clinical spectrum of cardiac manifestations of GPA, including the pathophysiology of valvular rupture and small-vessel coronary vasculitis. Large prospective registries and multicenter cohort studies are needed to identify predictors of cardiac complications, inform risk stratification, and refine the treatment algorithms. Comparative studies evaluating surgical versus medical management of valvular lesions, as well as long-term outcomes associated with biological therapies, will be crucial to guide care. Strengthening awareness of these atypical presentations and addressing current knowledge gaps will be vital to enhance early recognition, support individualized treatment decisions, and ultimately reduce the morbidity and mortality associated with complex systemic vasculitis.

## References

[1] Rout P, Garlapati P, Qurie A (2025). Granulomatosis with polyangiitis. StatPearls.

[2] Robson JC, Grayson PC, Ponte C (2022). 2022 American College of Rheumatology/European Alliance of Associations for Rheumatology classification criteria for granulomatosis with polyangiitis. Ann Rheum Dis.

[3] Klemmer PJ, Chalermskulrat W, Reif MS, Hogan SL, Henke DC, Falk RJ (2003). Plasmapheresis therapy for diffuse alveolar hemorrhage in patients with small-vessel vasculitis. Am J Kidney Dis.

[4] Pugnet G, Puéchal X, Fillatre P (2015). FRI0264 cardiac manifestations of granulomatosis with polyangiitis at diagnostic. Ann Rheum Dis.

[5] Da Silva RC, Adhikari P (2022). Granulomatosis with polyangiitis presenting with diffuse alveolar hemorrhage: a systematic review. Cureus.

[6] Walsh M, Merkel PA, Peh CA (2020). Plasma exchange and glucocorticoids in severe ANCA-associated vasculitis. N Engl J Med.

[7] Hellmich B, Sanchez-Alamo B, Schirmer JH (2024). EULAR recommendations for the management of ANCA-associated vasculitis: 2022 update. Ann Rheum Dis.

[8] Cocco G, Gasparyan AY (2010). Myocardial ischemia in Wegener's granulomatosis: coronary atherosclerosis versus vasculitis. Open Cardiovasc Med J.

[9] Dewan R, Trejo Bittar HE, Lacomis J, Ocak I (2015). Granulomatosis with polyangiitis presenting with coronary artery and pericardial involvement. Case Rep Radiol.

[10] Jeantin L, Lenfant T, Bataille P (2022). Antineutrophil cytoplasm antibody-associated vasculitides valvular impairment: multicenter retrospective study and systematic review of the literature. J Rheumatol.

[11] Uijtterhaegen G, De Donder L, Ameloot E, Lefebvre K, Van Dorpe J, De Pauw M, François K (2020). Aortic valve replacement due to granulomatosis with polyangiitis: a case series. Eur Heart J Case Rep.

[12] Al-Kindi SG, Amer Al-Aiti M, Yang M, Josephson RA (2017). Granulomatosis with polyangiitis presenting with acute aortic and mitral regurgitation: case report and big-data analysis. J Heart Valve Dis.

[13] Borowiec A, Rosinska M, Kowalik I, Rybski S, Chwyczko T, Jankowski J, Życińska K (2024). Cardiac valvular involvement in granulomatosis with polyangiitis in long-term observation. Rev Port Cardiol.

[14] Pichler Sekulic S, Sekulic M (2021). Case report: isolated and focal non-necrotizing granulomatous inflammation of mitral valves: a report of two cases. Front Cardiovasc Med.

[15] Marquetand C, Lamprecht P, Dressler FF (2020). GPA-induced granulomatous endocarditis mimicking a thrombotic mitral valve stenosis. JACC Case Rep.

[16] Kawada S, Kuriyama M, Tanabe A, Kioka Y (2015). Granulomatosis with polyangiitis (Wegener’s granulomatosis) complicated with ruptured posteromedial papillary muscle in the absence of coronary angiographic findings. Cardiology.

[17] Sahinoğlu I, Uslu S, Güc U, Can F, Ucar M, Tan A, Soysal Gündüz O (2025). Case report: mitral valve chordae tendineae rupture and splenic infarction in granulomatosis with polyangiitis. Int J Rheum Dis.

[18] Kim JS, Park YB, Lee SW (2023). Acute coronary syndrome in antineutrophil cytoplasmic antibody-associated vasculitis: a Korean single-centre cohort study. J Rheum Dis.

[19] Akhil N, Taksande A, Meshram RJ, Wandile S, Javvaji CK (2024). Exploring unusual cardiac complications: chorda tendinea rupture and pulmonary valve vegetation in infective endocarditis—a comprehensive review. Cureus.

[20] Ishikawa Y, Tokutsu K, Nakayamada S, Kuchiba A, Fushimi K, Matsuda S, Tanaka Y (2024). Short-term effectiveness and safety of rituximab versus cyclophosphamide for life-threatening ANCA-associated vasculitis: a propensity score analysis of the real-world nationwide database. Ann Rheum Dis.

[21] Jayne DR, Merkel PA, Schall TJ, Bekker P (2021). Avacopan for the treatment of ANCA-associated vasculitis. N Engl J Med.

[22] Sapkota N, Aryal Y, Basnet P (2025). Avacopan as a steroid-sparing therapy in relapsing granulomatosis with polyangiitis. Cureus.

[23] Pugnet G, Pagnoux C, Terrier B (2016). Rituximab versus azathioprine for ANCA-associated vasculitis maintenance therapy: impact on global disability and health-related quality of life. Clin Exp Rheumatol.

